# Bimekizumab for Darier disease: A case report highlighting dual IL-17A and IL-17F inhibition as a potential treatment

**DOI:** 10.1016/j.jdcr.2026.08.054

**Published:** 2026-09-08

**Authors:** Weipeng Xie, Priya Agarwal, Medha Rao, Lisa Zhou

**Affiliations:** a NYU Grossman School of Medicine, New York, New York; b Department of Dermatology, Rutgers Robert Wood Johnson Medical School, New Brunswick, New Jersey

**Keywords:** bimekizumab, bimzelx, case report, Darier disease, IL-17A, IL-17F, Interleukin-17 inhibitor

## Introduction

Darier disease (DD) is a rare autosomal dominant genodermatosis caused by mutations in the ATP2A2 gene, which encodes the sarco/endoplasmic reticulum calcium-ATPase 2 pump, leading to disruption of calcium homeostasis in keratinocytes.^1^, ^2^, ^3^ The estimated prevalence of DD is approximately 1 in 30,000–50,000 individuals, with onset typically occurring in the first or second decade of life. Clinically, DD is characterized by the formation of keratotic papules and plaques, predominantly in seborrheic areas (chest, back, scalp, forehead, and flexures), along with nail abnormalities (red and white longitudinal streaks, V-shaped notching), and mucosal involvement.^1^, ^2^, ^3^ Symptoms such as pruritus, burning, and malodorous secretions can significantly impair patients’ quality of life.^3^^,^^4^

The pathogenesis of DD involves not only calcium-mediated keratinocyte dysfunction but also a significant inflammatory component. Recent literature suggests that Th17-associated cytokines, such as interleukin-17 (IL-17) and IL-23, are involved in the pathogenesis of DD.^5^ Specifically, increased IL-17 A/F inflammatory pattern was observed in DD.^5^^,^^6^ Clinical support for IL-17 pathway inhibition in DD includes published reports demonstrating benefit with secukinumab (anti-IL-17A) and brodalumab (anti-IL-17RA) in adult patients, and IL-17A inhibition has also shown efficacy in pediatric DD.^5^^,^^7^^,^^8^

Bimekizumab (Bimzelx) is a humanized IgG1 monoclonal antibody with strong affinity for both IL-17A and IL-17F, offering a broader blockade of the IL-17 axis.^9^ Its dual inhibition has yielded promising results in chronic immune-mediated conditions, such as psoriasis and psoriatic arthritis, which involve IL-17 cytokines in their pathogenesis.^9^^,^^10^ Here, we present a case demonstrating the clinical effectiveness of bimekizumab in a patient with DD refractory to multiple conventional therapies.

## Case presentation

A 26-year-old female presented with a 16-year history of biopsy-confirmed DD, primarily affecting the face and hands. The patient denied a family history of DD. Her disease course was complicated by recurrent superficial skin infections requiring both topical and oral antibiotic therapy. She reported disease flares associated with heat exposure and during her monthly menstrual cycle. The extent of disease involvement and symptom burden significantly impaired her quality of life (Fig 1).

**Fig 1:**
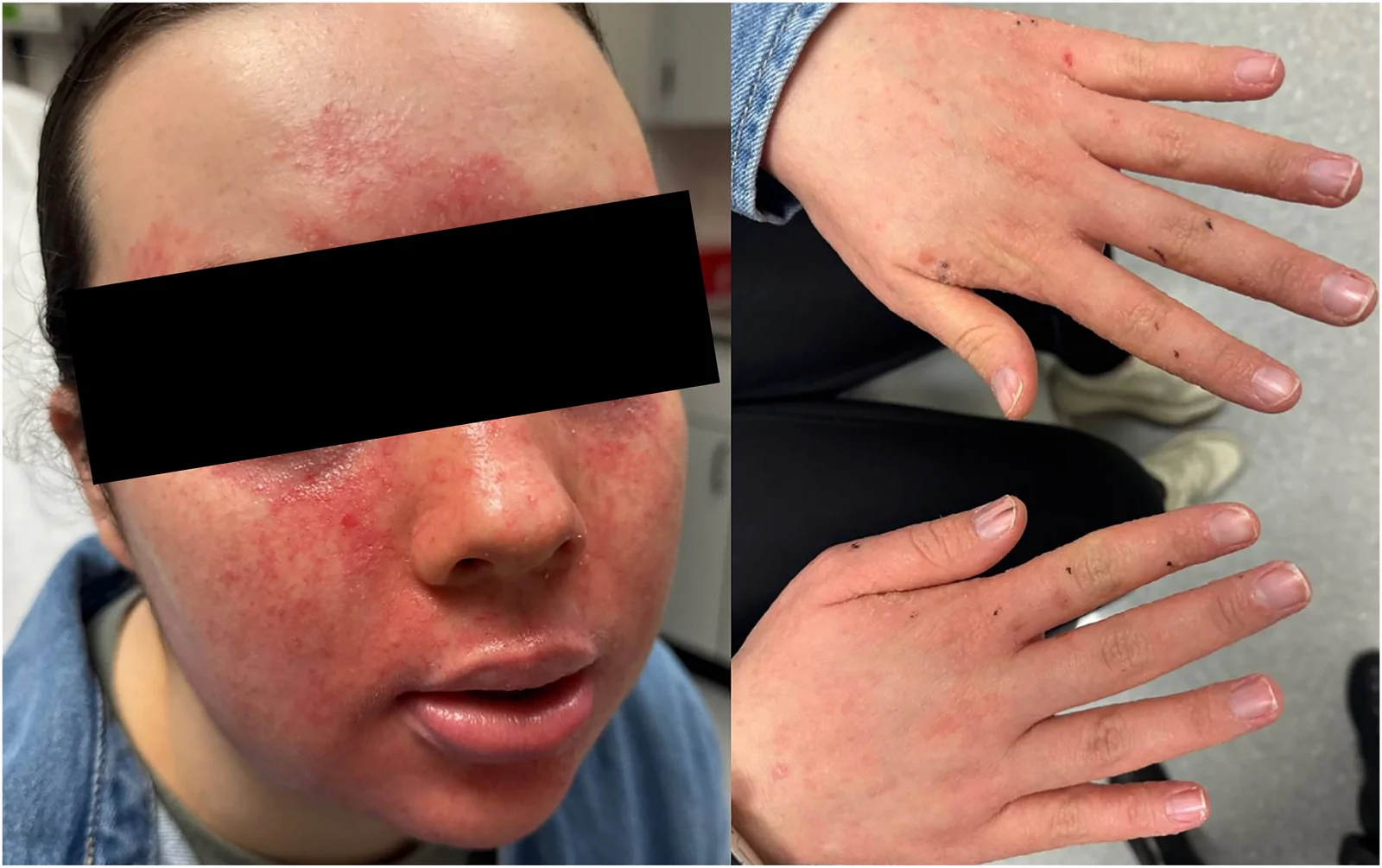
Manifestations of Darier disease in the patient. Patient’s initial presentation demonstrating erythematous scaling with flaking diffusely on the face and dorsal hands.

Prior to initiating bimekizumab, the patient had been treated with topical desonide, petrolatum ointment, topical tretinoin, spironolactone, oral contraceptives, and intermittent courses of oral isotretinoin over several years, without achieving adequate disease control. Given the inadequate response to conventional therapies and emerging evidence supporting the use of IL-17 inhibition in DD, treatment with bimekizumab was initiated.

Bimekizumab was initiated at 320 mg subcutaneously every 4 weeks, with a planned loading regimen consisting of 5 doses administered at Weeks 0, 4, 8, 12, and 16, followed by maintenance dosing every 4 weeks. Following the first month of treatment, the patient reported significant improvement in facial erythema, pruritus, and scaling, with more modest improvement of the hands. At the 3-month follow-up, marked clinical regression of cutaneous lesions was observed (Fig 2). No significant adverse effects were reported, and bimekizumab was well tolerated throughout the treatment course.

**Fig 2:**
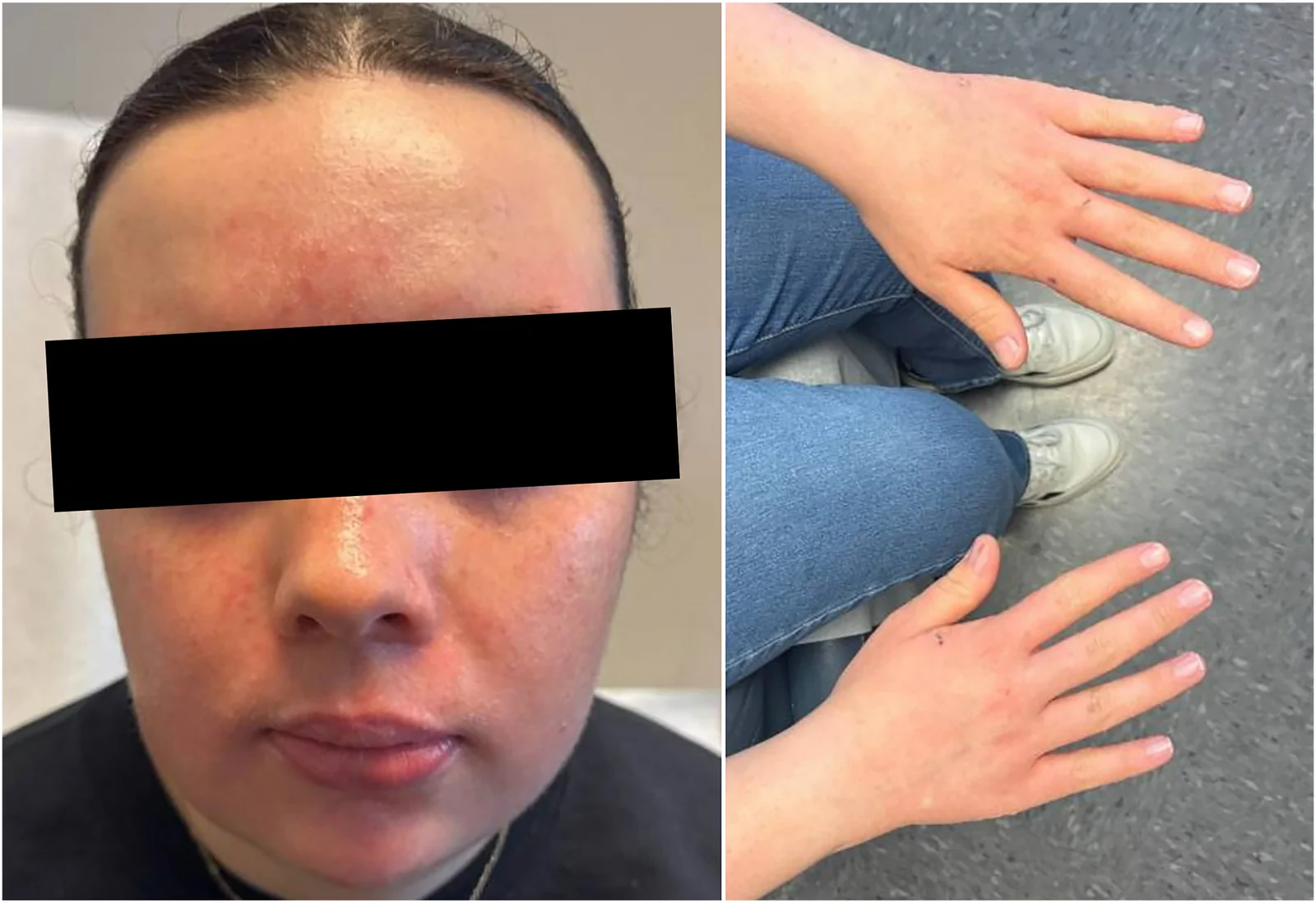
The patient after treatment with bimekizumab. Patient’s presentation following 3 doses of bimekizumab. Face demonstrates well-defined diffuse erythema with minimal scaling, significantly improved from the initial presentation. Monomorphic, erythematous, thin scaly papules were noted on the dorsum of the bilateral hands, moderately improved from the initial presentation.

## Discussion

This case report adds to the current evidence supporting IL-17 pathway inhibition as a therapeutic target in DD and demonstrates the potential of bimekizumab to effectively manage DD. Preclinical and clinical data indicate that IL-17A and IL-17F share overlapping but nonredundant roles: while IL-17A is the more potent inducer of keratinocyte inflammatory gene programs, IL-17F contributes substantially to neutrophil recruitment and further amplification of proinflammatory cytokine cascades.^9^

In a head-to-head phase 3b trial (BE RADIANT), bimekizumab demonstrated superior skin clearance compared to secukinumab (selective anti-IL-17A) in plaque psoriasis, with significantly higher rates of complete skin clearance (PASI 100) at Week 16 (61.7% vs. 48.9%) and maintained superiority at Week 48.^11^ Bimekizumab has demonstrated sustained, clinically meaningful improvements in difficult-to-treat disease subtypes including scalp, nail, and palmoplantar psoriasis over 4-year follow-up data.^12^ This clinical superiority supports the biological premise that IL-17F co-inhibition confers additional therapeutic benefit, which may extend to DD where both IL-17 A/F are upregulated in lesional skin.^5^^,^^6^ One important potential adverse effect of bimekizumab is the higher incidence of oral candidiasis compared to secukinumab, a selective IL-17A inhibitor.^11^ Clinicians considering bimekizumab for DD should proactively counsel patients about this risk, perform baseline oral assessment, and maintain a low threshold for antifungal therapy.

## Conclusion

Bimekizumab represents a mechanistically novel approach to treating DD by dual inhibition of IL-17A and IL-17F. This case suggests it may be a viable option for patients who have failed or cannot tolerate conventional therapies, including oral retinoids. The broader IL-17 axis blockade of bimekizumab compared to selective IL-17A inhibitors warrant further investigation. Larger prospective studies or case series can further confirm these findings.

### Declaration of generative AI and AI-assisted technologies in the writing process

None.

## Conflicts of interest

None disclosed.
